# Study on HOXBs of Clear Cell Renal Cell Carcinoma and Detection of New Molecular Target

**DOI:** 10.1155/2021/5541423

**Published:** 2021-07-08

**Authors:** Guangzhen Wu, Xiaowei Li, Yuanxin Liu, Quanlin Li, Yingkun Xu, Qifei Wang

**Affiliations:** ^1^Department of Urology, The First Affiliated Hospital of Dalian Medical University, Dalian 116011, China; ^2^Department of Urology, Shandong Provincial Hospital, Cheeloo College of Medicine, Shandong University, Jinan 250021, China

## Abstract

Our study examined the transcriptional and survival data of HOXBs in patients with clear cell renal cell carcinoma (ccRCC) from the ONCOMINE database, Human Protein Atlas, and STRING website. We discovered that the expression levels of HOXB3/5/6/8/9 were significantly lower in ccRCC than in normal nephritic tissues. In ccRCC, patients with a high expression of HOXB2/5/6/7/8/9 mRNA have a higher overall survival (OS) than patients with low expression. Further analysis by the GSCALite website revealed that the methylation of HOXB3/5/6/8 in ccRCC was significantly negatively correlated to gene expression, while HOXB5/9 was positively correlated to the CCT036477 drug target. As DNA abnormal methylation is one of the mechanisms of tumorigenesis, we hypothesized that HOXB5/6/8/9 are potential therapeutic targets for patients with ccRCC. We analyzed the function of enrichment data of HOXBs in patients with ccRCC from the Kyoto Encyclopedia of Genes and Genomes pathway enrichment and the PANTHER pathway. The results of the analysis show that the function of HOXBs might be associated with the Wnt pathway and that HOXB5/6/8/9 was coexpressed with multiple Wnt pathway classical genes and proteins, such as MYC, CTNNB, Cyclin D1 (CCND1), and tumor protein P53 (TP53), which further confirms that HOXBs inhibit the growth of renal carcinoma cells through the Wnt signaling pathway. In conclusion, our analysis of the family of HOXBs and their molecular mechanism may provide a theoretical basis for further research.

## 1. Introduction

Morbidity due to renal carcinoma is the third most common among urinary tumors [1] and increases annually. It is higher in males than in females [2]. The onset of renal carcinoma is unremarkable, with no specific symptoms and signs in the early stage, thus making an early diagnosis difficult. Moreover, metastases occur in approximately 20–30% of patients at the time of diagnosis. Conventional treatment methods for metastatic renal carcinoma have reportedly poor outcomes [3, 4]. Recently, a number of new methods have been introduced, such as targeted therapy and immunotherapy. Despite their effectiveness, these still have limitations. The administration of drugs gradually increases clinical drug resistance [2, 3]. Therefore, new indicators for clinical diagnosis and prognosis and therapeutic targets for renal cell carcinoma are urgently needed. On the etiology and pathogenesis of tumors, Reya and Clevers [5] concluded that tumors may result from abnormal organogenesis as DNA methylation could regulate the opening and closing of gene expression, which is associated with many diseases and physiological processes such as tumorigenesis and embryonic development. More interestingly, the protein encoded by HOXB acts as a nuclear transcription factor in vertebrate embryonic development, which codes for limb formation, regulates the genitourinary system, and specifies species diversity in the anterior and posterior axes of fetal bodies [6].

At present, it is well established that the composition of the HOXB cluster is highly conserved among all vertebrates, and the HOXB cluster is composed of B1–B9 and B13. HOXB genes all contain homeoboxes, with 70%–80% homology between genes. They have the ability to bind DNA and thus participate in the regulation of gene expression. However, HOXB has different effects on each tumor species. For example, in lung cancer, HOXB9, as a classical Wnt/TCF signaling pathway target, can significantly improve the invasion and metastatic potential of tumor cells and directly mediate the brain metastasis of lung adenocarcinoma [7]. HOXB2 can promote the metastasis of NSCLC by regulating the metastasis-related genes and serve as an index when judging the prognosis of stage I lung cancer. In gastrointestinal cancer, there is a stage differential HOXB13 expression in esophageal cancer. The expression of HOXB13 is increased in the submucosa or muscular layer, until the cancer tissue infiltrates into the adventitia [8]. HOXB9 has a low expression trend in gastric carcinoma. The more inferior expression, the lesser the differentiated degree of cancer tissue, the earlier the lymph node metastasis, and the shorter the OS of patients. Therefore, the low expression of HOXB9 may be an independent risk factor for poor prognosis of gastric carcinoma [9]. HOXB3/8/9 significantly induce colorectal carcinoma of the ascending colon. In breast cancer, HOXB7/9 could transform human mammary epithelial cells MCF10A and induce epithelial-mesenchymal transition (EMT).

Moreover, the high expression of HOXB9 reduces the disease-free survival rate of patients with breast cancer, which may be useful as an independent prognostic factor [10, 11]. In ovarian cancer, HOXB7 can enhance cell proliferation by promoting ovarian cancer cells to secrete more bFGF. HOXB7/13 can improve the invasion and metastatic potential of tumor cells. HOXB13 can also resist tamoxifen-induced apoptosis of cancer cells [12] and can inhibit the growth of prostate cancer cells through the Wnt signal pathway and androgen receptor signal pathway [13]. In other tumors, the functional diversity of HOXB also shows that HOXB7 could promote the progression and metastasis of pancreatic cancer [14]; metastasis and poor prognosis of bladder cancer are closely associated with the ectopic expression of HOXB13 in the cytoplasm [15].

In this study, we analyzed the expression and prognosis of HOXBs, family member molecules of ccRCCs, using data collected from the ONCOMINE database, Human Protein Atlas, STRING, and GSCALite. We found that expression levels of HOXB3/5/6/8/9 were significantly lower in ccRCC than in normal nephritic tissues. Furthermore, the expression level of the mRNA factor of HOXB2/5/6/7/8/9 in ccRCC was significantly associated with patient prognosis, comparatively, while that of HOXB3/4 was not. We used the STRING website to associate 40 genes with their closely related HOXBs. After that, we used the GSCALite to analyze the GO and the KEGG pathway analysis on 40 genes. In the end, we discovered that HOXBs participated in the embryonic development of living organisms.

## 2. Materials and Methods

### 2.1. ONCOMINE

ONCOMINE (https://www.oncomine.org/resource/main.html) is by far the largest cancer database, with the complete cancer mutation spectrum, gene expression database, and related clinical information, which is conducive for discovering new biomarkers and therapeutic targets. The ONCOMINE online database is used to analyze the mRNA expression levels of HOXBs in different tumor tissues.

### 2.2. GEPIA Dataset

GEPIA (http://gepia.cancer-pku.cn/) is a web page that integrates cancer data from The Cancer Genome Atlas (TCGA) and normal tissue data from the Genotype-Tissue Expression (GTEx) [16]. In this database, data can be collected more simply and quickly. We analyzed the differential expression of HOXBs in tumors, normal tissues, and different tumor stages. *P* < 0.05 and |log2FC| > 1 were considered statistically significant.

### 2.3. The Human Protein Atlas

The Human Protein Atlas (http://www.proteinatlas.org/) provides the distribution of human protein in human tissues and cells, using immunohistochemical technology to describe the distribution and expression of each protein in the body [17–19]. Through this database, we analyzed the OS of HOXBs in ccRCC and calculated the hazard ratio and 95% confidence interval. *P* < 0.05 was considered as statistically significant.

### 2.4. GSCALite Website

GSCALite (http://bioinfo.life.hust.edu.cn/web/GSCALite/) is a cancer genome analysis platform [20]. The report provided by GSCALite includes gene differential expression, overall survival, single nucleotide variation, copy number variation, methylation, pathway activity, miRNA regulation, normal tissue expression, and drug sensitivity. We used this online tool to explore the relationship between HOXBs and cancer pathways and finally to investigate their sensitivity to multiple anticancer drugs. *P* < 0.05 was regarded as statistically significant.

### 2.5. STRING Online Database

The STRING online database (https//string-db.org/) was used to analyze the relationship between HOXBs and screen genes that interact with HOXB [21, 22]. Through DAVID (https//david.ncifcrf.gov/home.jsp), we analyzed the GO, the KEGG pathway, and the PANTHER pathway. *P* < 0.05 was considered for statistical significance.

### 2.6. cBioPortal Analysis

The cBioPortal for Cancer Genomics (http://www.cbioportal.org/) provides molecular profiling and clinical prognostic relevance for cancer genome datasets. Using the cBioPortal online tool, we downloaded ccRCC with 537 samples (TCGA, Provisional) and used the CLUSTIVIS online tool for visualization. *P* < 0.05 was considered statistically significant.

## 3. Results

### 3.1. Transcriptional Levels of HOXB in Patients with RCC

**Figure 1:**
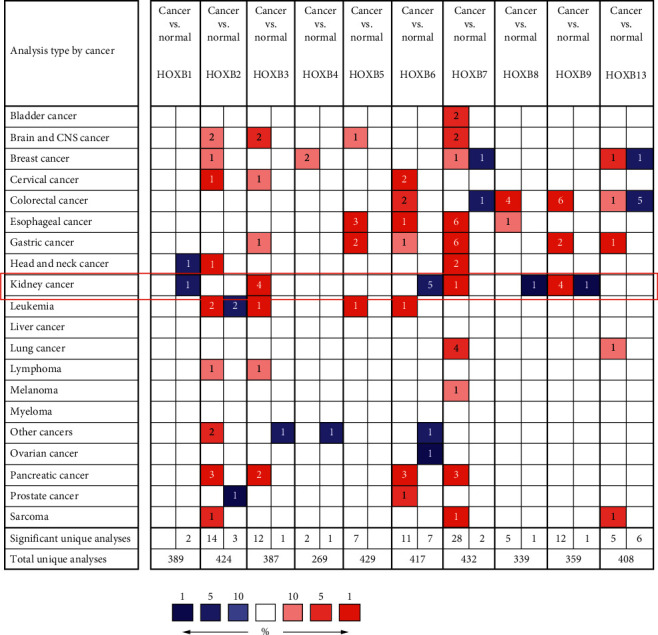
Comparison of the mRNA level of HOXBs in different tumor tissues by the ONCOMINE database. Among them, red represents high expression and blue represents low expression.

The ONCOMINE database is a classic oncogene chip database that integrates part of the data of the TCGA and GEO, aids in the screening of valuable target molecules or predicting phenotypes, and provides various analysis tools. This database allows visual display of analysis of cancer and normal tissue differential expressions and coexpression analysis and can be helpful in the analysis of drug sensitivity, mutation, or methylation-induced changes in gene expression. In this study, we analyzed the mRNA levels of HOXBs in tumors and normal tissue and found that the mRNA levels of HOXB1/6/8/9 are significantly downregulated in renal cell carcinoma, while those of HOXB3/4/7 are upregulated in nearly all the tumors, including renal cell carcinoma. In addition, HOXB2/7 have an obvious high expression of mRNA in a variety of tumors (Figure 1).

### 3.2. Relationship between the mRNA Level of HOXBs and the Clinicopathological Characteristics of Patients with RCC

**Figure 2:**
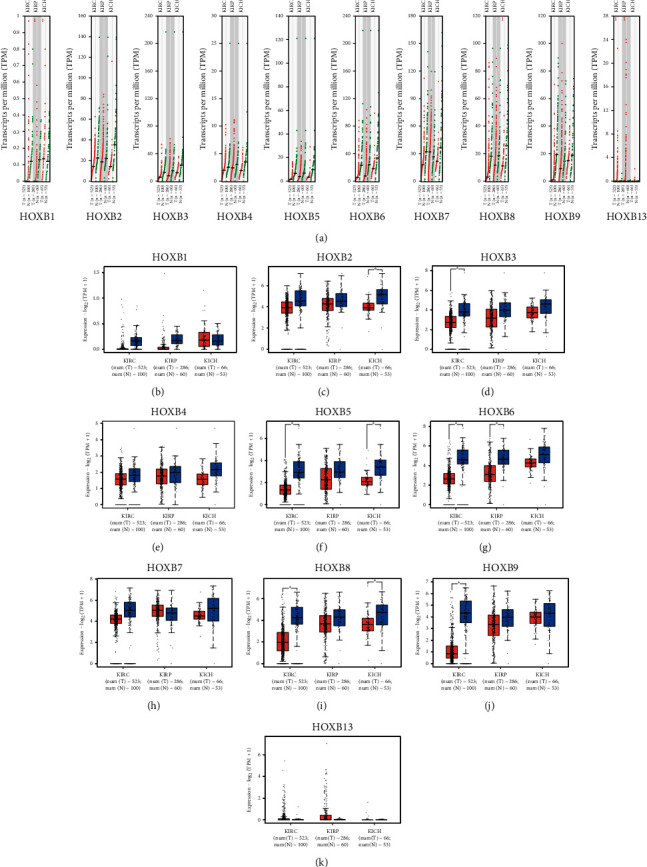
Expression of HOXBs in renal carcinoma (GEPIA). (a) The differential expression of HOXBs between normal kidney and KIRC, KIRP, and KICH using the GEPIA database. (b–k) Scatterplot of the differential expression of HOXBs between normal kidney and KIRC (kidney renal clear cell carcinoma), KIRP (kidney renal papillary cell carcinoma), and KICH (kidney chromophobe) using the GEPIA database (histogram). GEPIA, expression profile analysis.

**Figure 3:**
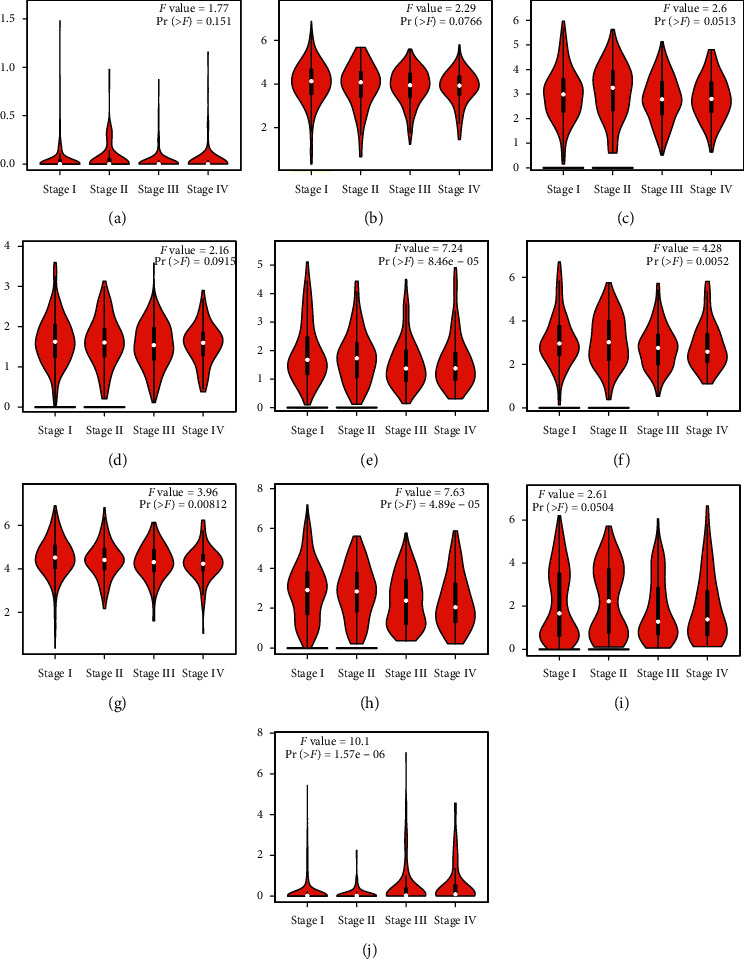
The differential expression of HOXBs in different stages of renal cell carcinoma using GEPIA database (a–j).

We used the GEPIA dataset to analyze the 10 genes of HOXB in patients with kidney cancer. In comparison with normal renal tissues, the expressions of HOXB3/5/6/8/9 in KRC, HOXB5/8 in KICH, and HOXB6 in KIRP were significantly downregulated (Figures 2(a)–2(k)). Because the morbidity of ccRCC is as high as 75% in all types of nephritic carcinoma [23] and HOXB shows a significant difference in ccRCC, we decided to primarily investigate ccRCC and analyzed the relationship between the expression of HOXBs and tumor stage in ccRCC. Significant differences were observed in HOXB5, HOXB6, HOXB7, and HOXB8 groups but not in HOXB1, HOXB2, HOXB3, HOXB4, and HOXB9 groups (Figures 3(a)–3(j)).

### 3.3. Difference of HOXBs Protein Levels between ccRCC and Normal Kidney Tissues and Their Relationship with Prognosis

**Figure 4:**
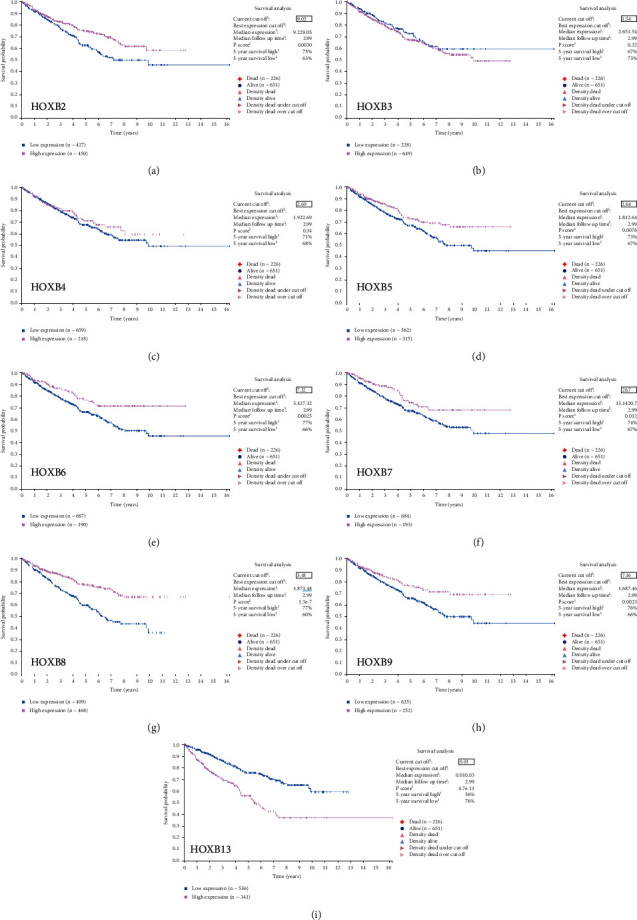
Using Human Protein Atlas to investigate the relationship between the mRNA of HOXBs and the prognosis (a–i). The expression of HOXB1 was too low to be analyzed.

To investigate different protein levels of HOXBs in patients with ccRCC and those with normal renal tissues, we used the Human Protein Atlas to verify the differential expression of HOXBs. We found that patients with a high mRNA level of HOXB2/5/6/7/8/9 have a higher OS, but it was not significantly different from that of HOXB3/4 (Figures 4(a)–4(i)).

### 3.4. Interactions, Copy Number Variation, Methylation, Cancer Potential Pathways, and Drug Sensitivity of Patients with ccRCC

**Figure 5:**
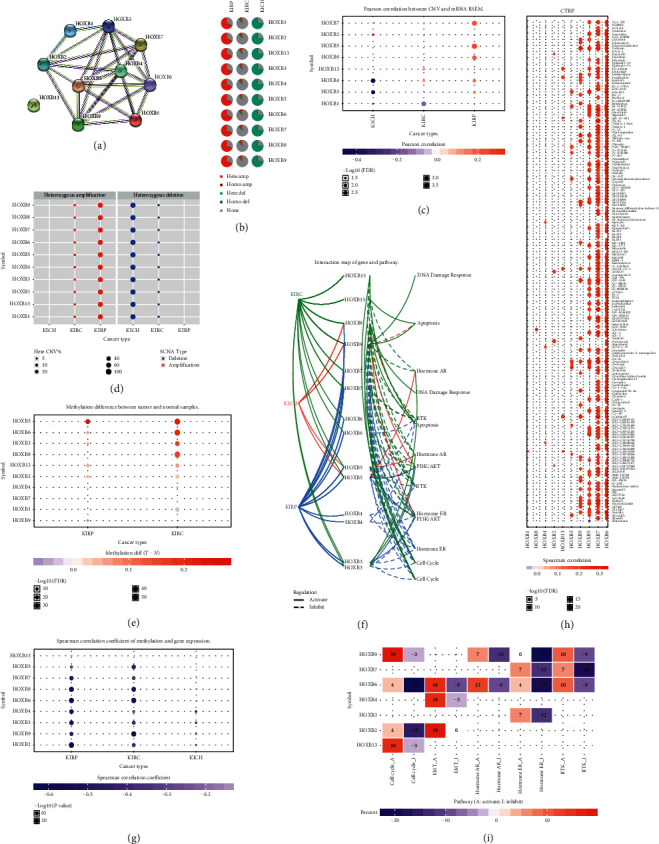
(a) Using the STRING website to analyze the interaction between HOXBs. (b–d) Using the GSCALite website to analyze the CNV of HOXBs. (e, g) Analyzing the DNA methylation of HOXBs. (f, i) Analyzing the potential pathway of HOXBs. (h) Analyzing the potential drug target of HOXBs.

Using the STRING online database to analyze the relationship between HOXBs, we found a close relationship between HOXB1-9 and the other genes and none with HOXB13 (Figure 5(a)). The expression of HOXs in tumor samples is significantly different from that of the normal renal tissues [24], and there may be variations in DNA fragments. Using the GSCALite to analyze the copy number variation (CNV), cancer potential pathways, and drug sensitivity, we found significant differences in the expression of CNV among KIRC, KIRP, and KICH. In KIRC, CNV amplification and deletion occur in a small number of heterozygotes. CNV amplifications and deletions occur in a large number of heterozygotes in KIRP and KICH, respectively (Figures 5(b) and 5(d)). We also found that the relevance between CNV and mRNA expression of HOXBs in RCC was different. In KICH, HOXB3/4 and HOXB1 CNV were negatively correlated to mRNA expression, while HOXB5/7/9 CNV was positively correlated to the same (Figure 5(c)). DNA methylation aberrance is the primary transcriptional regulation mechanism of tumorigenesis [25, 26].

In our study, the methylation of HOXB in KIRC and KIRP was upregulated compared to that of normal renal tissue. Also, in KIRC, KIRP, and KICH, the methylation of HOXB3/5/6/8 was significantly negatively correlated to the expression (Figures 5(e) and 5(g)). We found that the expressions of HOXBs were different in three types of renal cancers, but all showed an anticancer effect. Using the GSCALite to analyze the cancer potential pathways of HOXBs, we found that HOXB9 promotes DNA damage response, HOXB9/13 promote cell apoptosis, HOXB6/7 suppress PI3K/AKT classic cancer pathway, HOXB4/5/6/7/9 suppress receptor tyrosine kinase (RTK) pathway, HOXB3/5 promote hormone androgen receptor (AR), HOXB3/4/5/9 suppress hormone estrogen receptor (ER), HOXB2/6 suppress cell cycle, HOXB3/6/7/9 suppress hormone ER, and HOXB7 suppresses RTK pathway. Interestingly, HOXB2/4/6 are also inducers of EMT (Figures 5(f) and 5(i)). In our analysis of the relationship between HOXBs and a variety of traditional tumor drug targets, we found that HOXB5/7/9/13 were positively correlated to CCT036477 drug targets; HOXB7/8/9/13 were closely correlated to tretinoin, KW-2449, doxorubicin, and SB-225002; and HOXB9 was positively correlated to PX-12 drug activity. CCT036477 is a classical Wnt signal inhibitor that inhibits the growth of various cancer cells and the development of embryos and the expression of Wnt target genes (Figure 5(h)).

We used diverse databases to comprehensively analyze the expression of HOXBs and their relationship with OS. We discovered that the mRNA levels of HOXB1/6/8/9 were significantly downregulated in renal cell carcinoma, while HOXB3/4/7 were upregulated in almost all tumors, including RCC. Also, the high mRNA levels of HOXB2/5/6/7/8/9 were predicted to have a higher OS in patients, and there was no significant statistical difference in HOXB3/4. Therefore, we considered that HOXB 5/6/8/9 could be used as a potential target for ccRCC.

### 3.5. Coexpression of HOXBs in Patients with ccRCC

**Figure 6:**
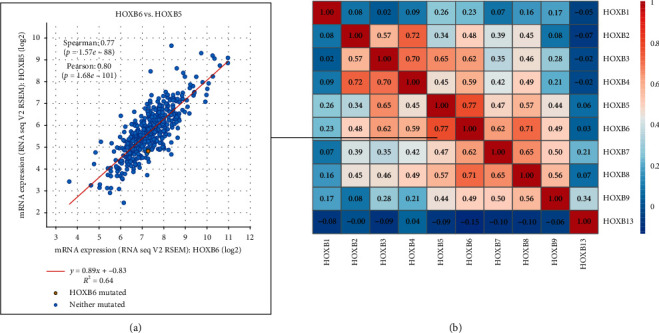
The coexpression of HOXBs was analyzed using the cBioPortal online tool and visualized using an online tool (a, b).

We analyzed the HOXBs coexpression relation for ccRCC with 537 samples (TCGA, Provisional) through the cBioPortal online tool; and we used CLUSTIVIS online tool for visualization. We found a clear positive correlation between HOXB1-9, among which HOXB5 and HOXB6 had the strongest correlation (Figure 6(a)). The following showed significant positive correlation: HOXB2 and HOXB4; HOXB3 and HOXB4; HOXB5 and HOXB6; and HOXB6 and HOXB8 (Figure 6(b)).

### 3.6. Predicting the Function and Pathways of HOXBs and Their Adjacent Gene Alteration in Patients with ccRCC

**Figure 7:**
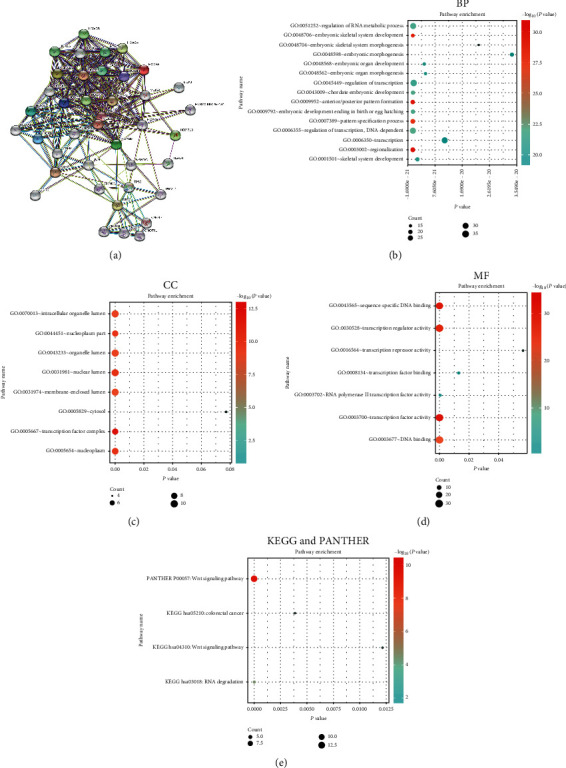
(a) Constructing a network of HOXB factors and their adjacent gene alteration through the STRING website. (b–d) Using the Gene Ontology analysis by DAVID tools, we predicted the functions of HOXBs and genes associated with HOXBs alterations and then visualized them through *R* language. (e) Through the analysis of KEGG and PANTHER using DAVID tools, predict the functions of HOXBs and genes associated with HOXB alterations and visualize them using the *R* language.

Through the STRING website, we linked 40 genes that are closely associated with HOXBs. Studying the genes' interaction, we constructed a network of 10 HOXBs and 40 genes with their frequently altered neighbor genes (Figure 7(a)) and then analyzed the enrichment of GO and KEGG and the PANTHER pathway through DAVID websites; the data were visualized through the *R* language.

Through GO analysis, we predicted, identified, and validated the function of the target gene from bioinformatics and expression system. We found that the change of HOXB in ccRCC has a significant regulatory effect on GO:0003002 (regionalization) (anterior/posterior) (pattern formation) and GO:0048706 (embryonic skeletal system development), which is beneficial to prove that HOXBs are associated with the development of biological embryos. At the same time, we found that GO:0043565 (sequence-specific DNA binding), GO:0030528 (transcription regulatory activity), GO:0003700 (transcription factor activity), and GO:0003677 (DNA biding) were also significantly controlled by HOXBs. Moreover, the PANTHER pathway analysis was visualized using the *R* language. We discovered that the overall genes were closely associated with TP53 and MEIS1, but, interestingly, HOXBs were also closely associated with c-JUN, PBX1, and CCNP1 (Figures 7(b)–7(d)).

**Figure 8:**
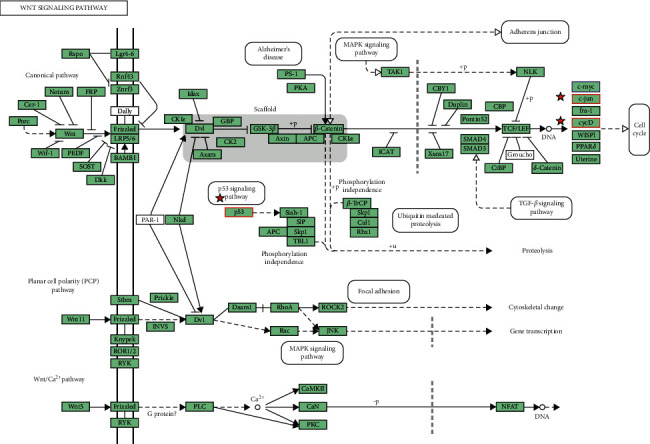
The Wnt pathway. The red mark indicates enrichment through the analysis of the KEGG pathway of the functions of HOXBs and genes associated with HOXBs alterations on the DAVID website.

The results analyzed by KEGG show that three pathways were associated with the change of HOXB function in ccRCC: Wnt signaling, colorectal cancer, and RNA degradation (Figure 7(e)). In these pathways, the Wnt signaling pathway is mainly responsible for the occurrence and development of ccRCC (Figure 8). Simultaneously, it is also confirmed that HOXBs are closely associated with cytokine transcription (Figures 7(b)–7(d)). This finding is consistent with Giampaolo A's findings [27].

### 3.7. Potential Mechanisms and Signaling Pathways of HOXBs

**Figure 9:**
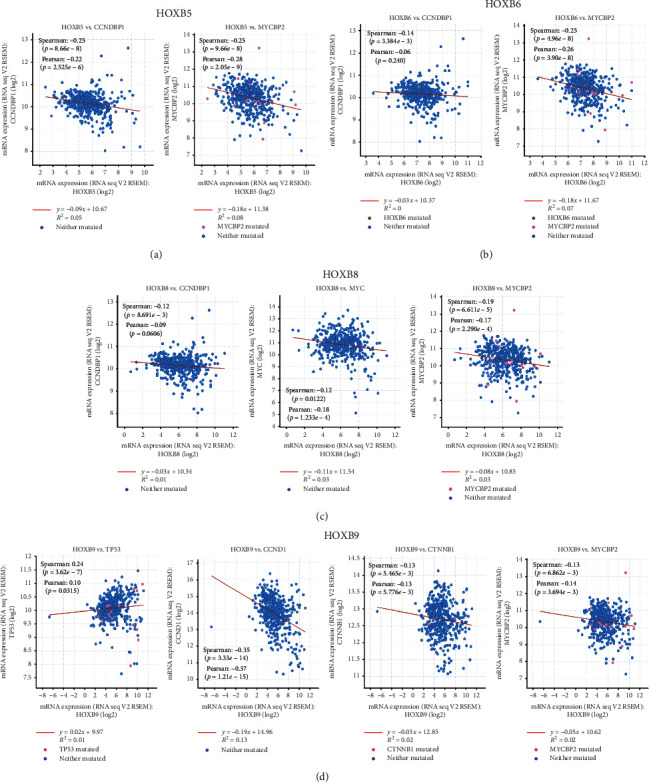
Through the cBioPortal website, we investigated the coexpression of the Wnt pathway classical protein with genes that have significance in expression and survival (a–d).

It is found that CCND1, CCNDBP1, MYCBP2, MYC, CTNNB1, *β*-Catenin, and TP53 are the classical proteins and genes of the Wnt pathway [28, 29]. We analyzed protein-protein coexpression on the HOXBs and Wnt pathway. Using the cBioPortal database, we discovered that HOXB5 is negatively associated with CCNDBP1 and MYCBP2; HOXB6 is negatively correlated with CCNDBP1 and MYCBP2; HOXB8 is negatively correlated with CCNDBP1, MYCBP2, and MYC; HOXB9 is negatively correlated with CCNDBP1, MYCBP2, and CTNNB1 and positively correlated with TP53 (Figures 9(a)–9(d)). In our study, HOXB5/6/8/9 can be used as a potential treatment target for ccRCC.

## 4. Discussion

Although we have partly confirmed the effect of HOXBs in the development and prognosis of various cancers [30, 31], there has not been an in-depth study on HOXBs in ccRCC. Studies have shown that bioinformatics analysis is a method that can help us quickly find biomarkers in the development of diseases [32–35]. Our study deeply investigates the expression and prognosis of multiple HOXBs in ccRCC. We hope that our research will help discover the effect of HOXBs, improving the treatment design and accuracy of prediction for ccRCC.

In this study, we examined the transcriptional and prognosis data of HOXBs in patients with ccRCC from the ONCOMINE database, Human Protein Atlas, and STRING website. We discovered that the expression levels of HOXB3/5/6/8/9 were significantly lower in ccRCC than in normal nephritic tissues. Furthermore, the expression level of mRNA factor of HOXB2/5/6/7/8/9 in ccRCC was related considerably to patient prognosis. A high level of the mRNA factor had a better OS in patients, comparatively, while HOXB3/4 did not. We studied the GSCALite website's analysis that the methylation of HOXB3/5/6/8 in ccRCC was significantly negatively correlated with gene expression. While HOXB5/9 were positively correlated with the CCT036477 drug target, CCT036477 was the classical Wnt signal inhibitor; and DNA abnormal methylation is one of the mechanisms of tumorigenesis. Therefore, our study implied that HOXB5/6/8/9 are potential therapeutic targets for patients with ccRCC. We examined the function of enrichment data of HOXBs in patients with ccRCC from KEGG enrichment and the PANTHER pathway. We also found that the function of HOXBs might be associated with the Wnt pathway. In coexpression analysis, we discovered that HOXB5/6/8/9 were coexpressed with multiple Wnt pathway classical genes and proteins, such as MYC, CTNNB, CCND1, and TP53, which further confirms that HOXBs inhibit the growth of renal carcinoma cells through the Wnt signaling pathway.

In conclusion, our systematic analysis of all members of HOXBs and their underlying molecular mechanisms may provide a basis for future research. Wnt/*β*-Catenin signaling pathway activated EMT [36] to promote the binding of *β*-Catenin to the nucleus and T cell-specific transcription factor/lymphoenhancer (TCF/LEF) to regulate the expression of E-cadherin, the characteristic factor of EMT, and then affect the occurrence of EMT, which plays a blocking role in the process of tumor invasion and migration. In other studies, it was found that HOXB5 protein could bind with its cofactor PBX1 to induce apoptosis, thus delaying tumor progression [37]. Our research found that HOXB5 inhibited the key genes CCND1 and MYC in the Wnt/*β*-Catenin signaling pathway, inhibited the tumor's occurrence, and inhibited the classical cancer pathway RTK pathway to inhibit cancer and improve OS. However, the expression of HOXB5 in different stages of RCC is significantly different, which suggests that HOXB5 plays a positive role in the early stage of RCC. We discovered that reducing the expression of HOXB5 in the late stage of ccRCC is more conducive to reducing the metastasis of RCC. This may be a direction for further research. We discovered that HOXB5 and HOXB6 are most closely related in HOXBs, similar to the regulatory mechanism of HOXB5. HOXB6 is negatively associated with MYC and CCND1. MYC and CCND1 are the critical genes in the Wnt signaling pathway and inhibit the RTK pathway, thus inhibiting independent downstream signaling pathways such as Ras/ERK or P13-K/Akt signaling pathway and thus inhibiting cell cycle and proliferation [38].

Interestingly, HOXB6 inhibited Wnt/*β*-Catenin signal pathway activation and enhanced EMT. It can be concluded that HOXB6 can inhibit cell proliferation but cannot control tumor invasion and migration. In this study, we found that the expression of HOXB8 in normal kidney tissue was significantly different from that in ccRCC. The low level of HOXB8 reduced the OS of ccRCC; however, we found that HOXB8 may also control tumor invasion by inhibiting CCND1 and MYC in the Wnt signaling pathway. However, there is no strong evidence.

We found that the expression of HOXB9 in renal carcinoma patients was lower than that in normal people. Besides, based on the Human Protein Atlas analysis, we discovered that the higher the expression of HOXB9 mRNA, the higher the OS. At the same time, HOXB9 had a significant negative correlation with MYC, CTNNB1, CCND1, and their expression proteins in the Wnt signaling pathway. We speculated that HOXB9 might block the Wnt signaling pathway and inhibit tumor growth through MYC and CTNNB1. Through the integration and transmission of information inside and outside the cell and then through the key genes of regulatory points, the cell decides whether to continue to divide, differentiate, undergo apoptosis, or enter the G0 stage to repair damaged DNA. There are many regulatory points of the cell cycle in the process from G1 to S. We found that HOXB9 was negatively correlated with CCND1. It may inhibit the cell cycle from the G1 phase to the S phase, inhibit transcription, regulate cell mitosis, and inhibit cell proliferation and immortalization by inhibiting the CCND1 coding protein Cyclin D1 binding cyclin-dependent kinase CDK4 [39]. At the same time, HOXB9 negatively regulates RTK, thus inhibiting the proliferation of cancer cells. In addition to inhibiting cancer cell proliferation, HOXB9 can activate TP53, regulate expression, promote cell apoptosis, inhibit growth, block the cell cycle, promote cell differentiation and DNA repair, maintain cell genome stability, and inhibit tumor angiogenesis [40]. Some studies have shown that androgen receptors can promote the metastasis of renal cell carcinoma. At the same time, HOXB9 can reduce the androgen receptor and promote the expression of cycHIAT1 by regulating the expression of the host (HIAT1) at the transcription level. As a result, regulating the expression of miR-195-5p/29A-3P/29C-3P can affect the migration and invasion of renal cell carcinoma [41]. In this study, we found that the inhibition of HOXB9 may be associated with the above pathways, and the above mechanisms will be verified in our future experiments.

There is no significant difference in expression of HOXB1/2/3/4/7 between normal kidney tissue and renal clear cell carcinoma in the current study. Therefore, we need to increase the sample size further or carry out experiments to verify the above results. Our research shows that the level of HOXB13 mRNA in ccRCC and renal tissue is almost no expression. However, HOXB13 is associated with various renal carcinoma targets involved in the Wnt pathway but combined with the introduction of HOXB13 through the Wnt signal pathway and the androgen receptor signal pathway to inhibit the growth of prostate cancer cells. We consider that although HOXB13 participates in the Wnt pathway, it may be different from the receptor of renal carcinoma. At the same time, HOXB13 is not significantly associated with other genes in the HOXB genome, so we speculate that HOXB13 may not be associated with renal cells' progress. At present, there are few studies on HOXBs in kidney cancer. We systematically analyze all members of HOXBs and their potential mechanisms. Our study may be used as a foundation for future research.

## 5. Conclusions

In summary, we discovered that HOXB5/6/8/9 are potential targets of precision therapy for patients with ccRCC. The role of HOXB5/6/8/9 in ccRCC may be associated with the Wnt pathway. However, our conclusion needs to be verified by further experiments, as there are few studies on HOXBs in kidney cancer at present. We have conducted a comprehensive analysis of all members of HOXBs and studied their molecular mechanism, thereby laying a foundation for future studies.

## Data Availability

The data used to support the findings of this study are available from the corresponding author upon request.

## Abbreviations

HOXB: Homeobox B
ccRCC: Clear cell renal cell carcinoma
OS: Overall survival
MYC: MYC protooncogene
CTNNB: Catenin beta
CCND1: Cyclin D1
TP53: Tumor protein P53
GO: Gene Ontology
KEGG: Kyoto Encyclopedia of Genes and Genomes
TCGA: The Cancer Genome Atlas
CNV: Copy number variations
KIRC: Kidney renal clear cell carcinoma
KIRP: Kidney renal papillary cell carcinoma
KICH: Kidney chromophobe
HIAT1: Hippocampus abundant transcript 1.
